# A case of juvenile eosinophilic cholangitis: Rapid peripheral blood hypereosinophilia after admission leading to diagnosis

**DOI:** 10.1002/jgh3.12454

**Published:** 2020-11-10

**Authors:** Shusaku Fukatsu, Katsuya Kitamura, Yasutsugu Asai, Kazumasa Nagai, Miho Kikuchi, Kyoko Asano, Kenichi Tadokoro, Fumito Yamanishi, Yusuke Tomita, Masakazu Abe, Takuya Wada, Yubu Matsue, Daisuke Nutahara, Junichi Taira, Hironori Nakamura, Takao Itoi

**Affiliations:** ^1^ Department of Gastroenterology and Hepatology Tokyo Medical University Hachioji Medical Center Tokyo Japan; ^2^ Department of Gastroenterology and Hepatology Tokyo Medical University Tokyo Japan

**Keywords:** bile duct stricture, eosinophilic cholangitis, hypereosinophilia

## Abstract

A 15‐year‐old boy was referred to our hospital with elevated hepatobiliary enzyme levels and jaundice. Magnetic resonance cholangiopancreatography performed at the previous medical facility revealed a stricture of the intrahepatic and extrahepatic bile duct. Computed tomography showed dilatation and wall thickness of the intrahepatic bile ducts. Primary sclerosing cholangitis or cholangiocarcinoma was suspected. Endoscopic retrograde cholangiopancreatography (ERCP) showed stricture in the intrahepatic and extrahepatic bile duct. On admission, the eosinophil count in the peripheral blood was normal; however, rapid hypereosinophilia in the peripheral blood was observed after admission, leading us to suspect eosinophilic cholangitis (EC). A bile duct biopsy showed inflammatory cells and eosinophil infiltration during a second ERCP. The patient was diagnosed with EC based on histopathology.

## Graphical abstract

Eosinophilic cholangitis was diagnosed based on the elevated blood eosinophil count after admission and bile duct pathology.

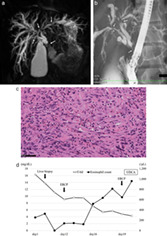

## Introduction

Eosinophilic cholangitis (EC) is rare and has no specific diagnostic criteria. However, it is often diagnosed by pathology or steroid responsiveness. Bile duct stricture due to eosinophilic infiltration underlies the pathogenesis of EC, although its relation to the peripheral blood eosinophil count is unknown. We report a case of juvenile EC that was suspected by rapid peripheral blood hypereosinophilia after admission and diagnosed by histopathology.

## Case report

A 15‐year‐old boy was referred to our hospital because of elevated hepatobiliary enzymes and abnormal imaging findings. One week prior, he presented to a nearby hospital with itching (lasting 1 month), jaundice (lasting 2 weeks), grayish white stool, and abdominal pain. He had no remarkable medical and family history; however, he reported food allergies to Rosales and peanuts. On admission, laboratory studies revealed an elevated hepatobiliary enzyme level, with a total bilirubin level of 22.6 mg/dL, direct bilirubin of 16.3 mg/dL, aspartic aminotransferase of 83 IU/L, alanine aminotransferase of 75 IU/L, and alkaline phosphatase of 897 IU/L. Peripheral blood eosinophilia was not observed. Hepatitis B surface antigen, hepatitis C virus antibody, serum tumor markers, and autoimmune antibodies were all negative. Serum immunoglobulin G (IgG) 4 level was within the normal range.

**Figure 1:**
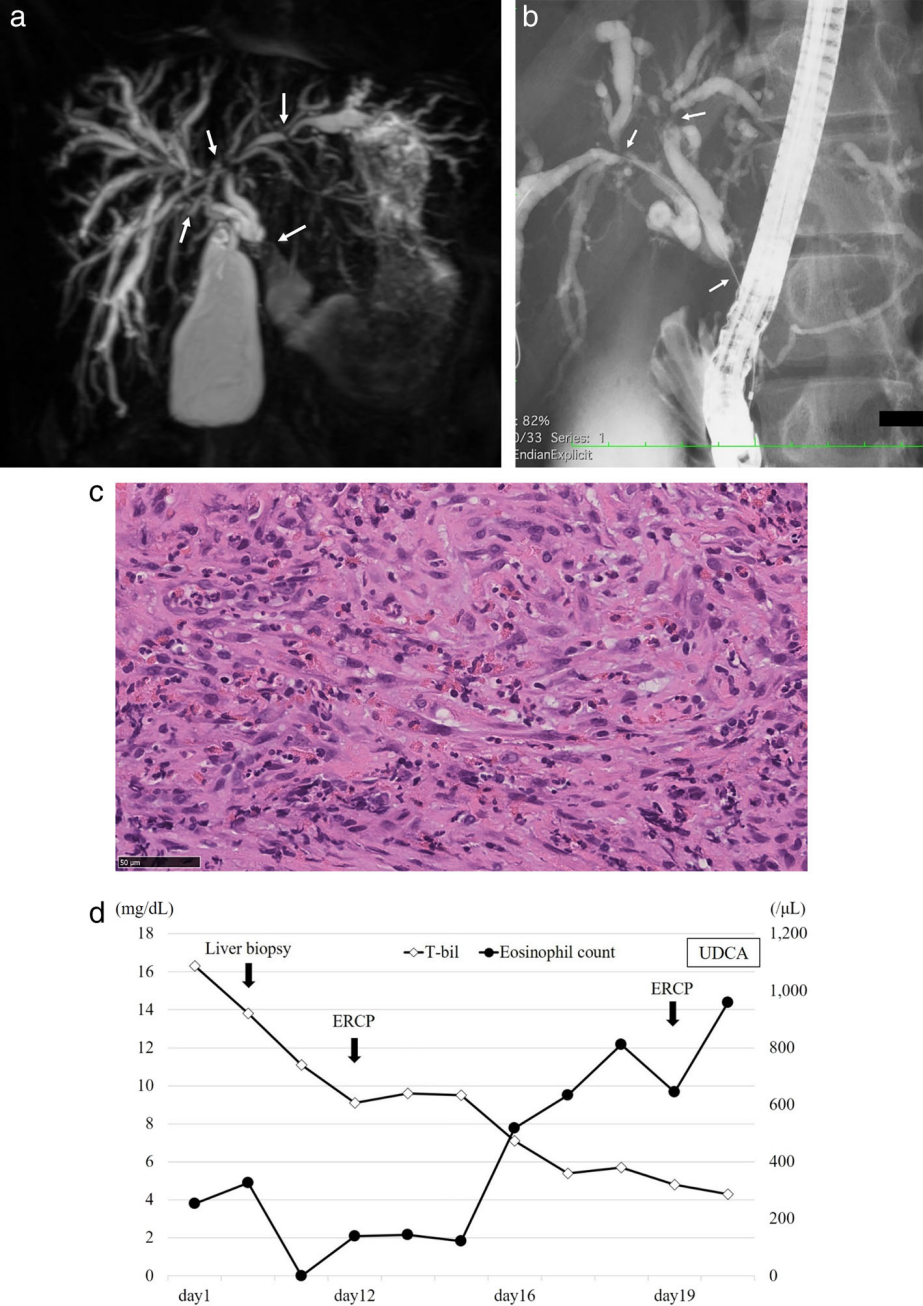
(a) The magnetic resonance cholangiopancreatography (MRCP) from the previous hospital. (b) The first endoscopic retrograde cholangiopancreatography (ERCP) findings. (c) The bile duct biopsies findings. (d) The change in the blood levels of total bilirubin and eosinophil after admission.

Magnetic resonance cholangiopancreatography (MRCP) performed at the previous medical facility revealed a stricture of the intrahepatic and extrahepatic bile duct (Fig. 1a). Contrast‐enhanced computed tomography (CT) revealed dilatation and wall thickness of the intrahepatic bile ducts. To rule out primary sclerosing cholangitis (PSC), the patient underwent a liver biopsy on the fifth hospital day. The liver biopsy specimen showed no evidence of an onion‐skin lesion, a scar replacing the duct, nor malignancy. The patient then underwent upper and lower gastrointestinal endoscopy on the 10th hospital day. There was no evidence of inflammatory bowel disease (IBD); however, because of concerning ulcers on the terminal ileum and the ileocecal valve, the patient underwent intestinal biopsies. The intestinal biopsy specimens showed no evidence of IBD except for intestinal epithelial metaplasia. Furthermore, the patient underwent endoscopic retrograde cholangiopancreatography (ERCP) on the 12th hospital day for evaluation of bile duct strictures. ERCP findings revealed a stricture of the left and right intrahepatic and extrahepatic bile duct (Fig. 1b). A brush cytology of the extrahepatic bile duct stricture and endoscopic nasobiliary drainage was performed. Brush cytology revealed atypical cytology without evidence of malignancy. The patient underwent a second ERCP on the 19th day of hospitalization, and a biliary plastic stent was inserted after the extrahepatic bile duct stricture biopsy using normal forceps. The pathology showed no evidence of malignancy, although inflammation of infiltration cells mainly comprised lymphocytes, plasma cells, and eosinophils (at least 80 eosinophils per high‐power field [HPF]) (Fig. 1c). Immunohistochemical staining of the pathology revealed no marked infiltration by IgG4‐positive plasma cells. Peripheral blood eosinophils were elevated on the 16th hospital day, whereas they were within normal limits on admission (Fig. 1d), which caused us to suspect EC. We diagnosed EC based on the presence of hypereosinophilia and pathology findings of eosinophilic infiltration in the biliary epithelium. Because of the patient's young age, ursodeoxycholic acid (UDCA) with fewer side effects, rather than oral corticosteroids, was administered. The patient was discharged home with no events.

## Discussion

In our case, the increase in peripheral blood eosinophil count, which was within normal limits at admission, caused us to suspect EC. There is no clear association between EC and peripheral blood eosinophils; hence, this indicates the importance of continuous monitoring.

EC is a rare disease in which intrahepatic and extrahepatic bile ducts have stricture or dilatation due to infiltration of eosinophils into the bile ducts. The differential diseases include PSC; cholangiocarcinoma; IgG4‐related sclerosing cholangitis; and other causes of cholangitis such as intraductal stones, surgical or blunt abdominal trauma, and intra‐arterial chemotherapy.^1^ A literature search of “eosinophilic cholangitis” in PubMed for the period of 1997–2019 resulted in only 19 case reports. The median patient age was 49 years, the male‐to‐female ratio was 8:11, and 6 of 14 cases (43%) had elevated peripheral blood eosinophil counts (>1500/μL) (with a median of 862/μL) (Table 1).

**Table 1:** Outcomes of 19 patients searched for eosinophilic cholangitis

Age	Median (IQR), years	49 (36–68)
Gender	Male/female, *n*	8/11
Peripheral blood eosinophils	Median (IQR), /μL	862 (494–2447)
Location of biliary stricture	Hilar, *n*	9
	Diffuse, *n*	6
	Distal, *n*	4
Diagnostic method	Surgical pathology, *n*	11
	Bile duct biopsy or cytology, *n*	7
	Liver biopsy, *n*	1
Treatment	Surgical resection, *n*	9
	Oral corticosteroid, *n*	9
	Follow‐up, *n*	1

IQR, interquartile range.

EC is difficult to diagnose. Case management is difficult when a bile duct stricture is observed with imaging, such as in MRCP and ERCP, and a bile duct biopsy shows no evidence of PSC or malignancy due to cholangiocarcinoma; however, PSC or cholangiocarcinoma cannot be ruled out. EC can epidemiologically occur in relatively young people; thus, it is essential to avoid facile surgical resection. EC is relatively steroid‐responsive, and 9 out of 19 cases (47%) are treated with steroids alone (Table 1). Although steroids may be administered as a diagnostic treatment, the diagnosis may not be made if the steroid response is low.^2^

Eosinophils are associated with fibrosis and remodeling of various tissues, such as endomyocardial fibrosis and retroperitoneal fibrosis,^3^ suggesting that infiltration of eosinophils directly damages the bile ducts, causing fibrosis and bile duct stricture.

If the pathogenesis of EC is assumed to be a direct result of eosinophilic infiltration, it is important to confirm eosinophilic infiltration by bile duct pathology for diagnostic purposes. The number of reports of bile duct biopsies or cytology that made a diagnosis of EC was small, and only seven cases were found in the reports (Table 1). Until recently, bile duct biopsy during endoscopy was challenging to perform, and this may be why the diagnosis was made only during surgical pathology. The number of infiltrated eosinophils in bile duct biopsies is not well documented (only three cases), and the number of infiltrated eosinophils ranges from 4 to 60 per HPF.^4^, ^5^, ^6^ Walter *et al*. reported that eosinophil infiltration in bile ducts in preoperative liver biopsies from liver transplantation donors averaged only about 0.1 cells per HPF,^7^ suggesting that eosinophil infiltration in the bile ducts of healthy individuals was almost nonexistent. Our patient was diagnosed with EC with at least 80 eosinophil infiltrations per HPF. It is crucial to understand the pathogenesis of bile duct biopsy and the extent of eosinophil infiltration in EC. Further studies should be conducted to qualify bile duct stricture biopsies using new devices.

There are no treatment guidelines for EC, although steroids and UDCA are often the treatments of choice. Because of our patient's young age, we chose to administer UDCA with fewer side effects. We will consider administering steroids if the bile duct stenosis findings do not improve in the future.
